# Priority setting of health interventions: the need for multi-criteria decision analysis

**DOI:** 10.1186/1478-7547-4-14

**Published:** 2006-08-21

**Authors:** Rob Baltussen, Louis Niessen

**Affiliations:** 1 Institute for Medical Technology Assessment (iMTA), ErasmusMC Rotterdam, Rotterdam, The Netherlands; 2 Department of Public Health, University Medical Centre Nijmegen, Nijmegen, The Netherlands; 3 Department of Health Policy and Management, ErasmusMC, Rotterdam, The Netherlands

## Abstract

Priority setting of health interventions is often ad-hoc and resources are not used to an optimal extent. Underlying problem is that multiple criteria play a role and decisions are complex. Interventions may be chosen to maximize general population health, to reduce health inequalities of disadvantaged or vulnerable groups, ad/or to respond to life-threatening situations, all with respect to practical and budgetary constraints. This is the type of problem that policy makers are typically bad at solving rationally, unaided. They tend to use heuristic or intuitive approaches to simplify complexity, and in the process, important information is ignored. Next, policy makers may select interventions for only political motives.

This indicates the need for rational and transparent approaches to priority setting. Over the past decades, a number of approaches have been developed, including evidence-based medicine, burden of disease analyses, cost-effectiveness analyses, and equity analyses. However, these approaches concentrate on single criteria only, whereas in reality, policy makers need to make choices taking into account multiple criteria simultaneously. Moreover, they do not cover all criteria that are relevant to policy makers.

Therefore, the development of a multi-criteria approach to priority setting is necessary, and this has indeed recently been identified as one of the most important issues in health system research. In other scientific disciplines, multi-criteria decision analysis is well developed, has gained widespread acceptance and is routinely used. This paper presents the main principles of multi-criteria decision analysis. There are only a very few applications to guide resource allocation decisions in health. We call for a shift away from present priority setting tools in health – that tend to focus on single criteria – towards transparent and systematic approaches that take into account all relevant criteria simultaneously.

## Background

**Figure 1:**
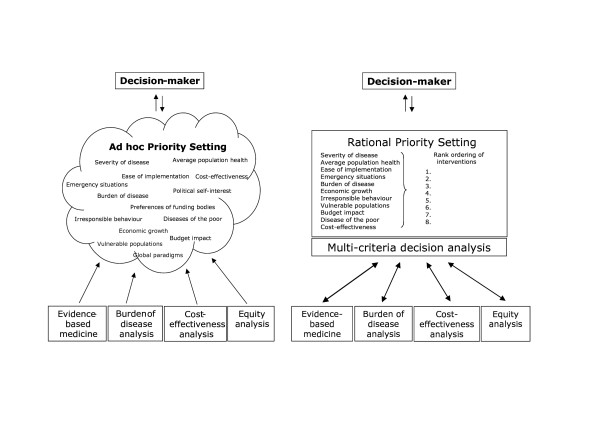
Ad hoc priority setting and rational priority setting.

Pertaining health needs and accelerating technological development put an ever-increasing demand on limited health budgets. Policy makers need to make important decisions on the use of public funds – to target which disease areas, which populations, and with which interventions. However, these choices may not be based on a rational and transparent process, and resources may not be used to an optimal extent [1,2]. For example, despite evidence that investing in primary health care is more effective than investing in specialized health care, allocations to primary care in Ghana have remained behind those allocated to tertiary care [3]. The underlying problem is that decisions on the choice of health interventions are complex and multifaceted [4,5], and the process is therefore ad-hoc or history-based [1,2]. Many criteria, or factors, play a role, and present the type of problem that behavioral decision research shows policy makers are typically quite bad at solving, unaided [6,7] (Figure 1).

A first, and probably most important, criterion is the societal wish to *maximize general population health*. This has indeed been the basis of many national disease programs in the past century [8]. A second set of criteria relates to the *distribution of health *in the population. Societies may give high priority to interventions that target vulnerable population groups such as the poor [9,10], the severely ill [11], or children or women of reproductive age [12], because they are more deserving of health care than others [13,14]. Also, societies may give high priority to the economically productive people to stimulate economic growth [15], or low priority to people who require health care as a result from irresponsible behavior (e.g. smoking) [16]. A third set of criteria responds to *specific societal preferences*, e.g. for acute care in life threatening situations, or for curative over preventive services [17].

A fourth set of criteria relates to the *budgetary and practical constraints *that policy makers face when implementing interventions, including costs and availability of trained health workers [18], and may take these into account when choosing between interventions. Fifthly, political criteria may play an important role. Policy makers may not always be benevolent maximizers of social welfare, but may also act out of own (political) self-interest [19]. Interests groups in societies exercise their influence on policy makers to prioritise interventions according to their objectives, and policy makers may be sensitive to this in their efforts to maximize political support. For example, health expenditures in many developing countries are often focused on services for richer areas or groups at the expense of the poor, even where the latter offers greater scope for cost-effective healthcare [19]. Also, policy makers may follow funding preferences of (international) organisations, which may not always cohere with national priorities [20-22]. The above list may not be exhaustive, and still other criteria may be important.

When confronted with such complex problems, policy-makers tend to use intuitive or heuristic approaches to simplify complexity, and in the process, important information may be lost, and priority setting is ad-hoc. Or worse, they act out of political self-interest and prioritize interventions according to their own objectives. In other words, policy makers may not always well placed to make informed well-thought choices involving trade-offs of societal values [6,7].

The above indicates the need for a rational and transparent approach to priority setting that guides policy makers in their choice of health interventions, and that maximizes social welfare. This paper presents an overview of the approaches that have been developed over the past decades, and argues that these offer little guidance to policy makers. They concentrate on single criteria only, whereas in reality, policy makers need to make choices taking into account multiple criteria simultaneously. Moreover, they do not cover all criteria that are relevant to policy makers. In other disciplines, multi-criteria decision analysis (MCDA) is routinely used in similar problems, and we show its basic concepts and most important methods. We call for the application of MCDA in health, and present some first examples.

## Rational approaches to priority setting

The past decades have witnessed the development of number of rational and transparent approaches to priority setting. Most prominent has been the development of *evidence-based medicine*, or the use of interventions with established effectiveness. This dates back to the beginning of the last century but was institutionalized by the foundation of the Cochrane Collaboration in 1993 [23-25]. The Cochrane Collaboration produces and disseminates systematic reviews of healthcare interventions and promotes the search for evidence in the form of clinical trials and other studies of interventions.

Because of steep increases in health interventions costs in western countries in the 1980's, economists proposed the use of *cost-effectiveness analysis *of health interventions. The underlying notion is that interventions should not only have established effectiveness, but should also be worth its costs [26]. For a certain budget, population health would then maximized by choosing interventions that show best value for money ('most cost-effective'). The World Bank promoted the concept in developing countries in 1993 [27] and recently the World Health Organization have made such information available at the regional level through the WHO-CHOICE project, e.g. on tuberculosis and HIV/AIDS control [28-30]. Work is underway to apply these cost-effectiveness estimates to the country level [31].

Also in the early 1990's, the World Bank expanded epidemiological mortality measures to the concept of *burden of disease analysis *[32]. Burden of disease analysis measures ill health in terms of morbidity and mortality to indicate the most important disease areas in a country. Its proponents consider the analysis as an important aid to priority setting as it would guide policy makers in targeting their intervention at the most important disease areas. Others argue that it lacks a conceptual basis for priority setting of health interventions, as the size of a disease problem has no relation to the potential for effective reduction [33]. Nevertheless, burden of disease analysis has been applied in many developed and developing countries including Eritrea, Kenya, Ethiopia, Uganda, and Tanzania in East Africa, Algeria, Morocco and Tunis in Northern Africa, and India [34,35].

With advances in population health in developing countries in the past decades, policy makers have increasingly become aware of disparities in health status between different groups in society. The past few years has witnessed an increased attention for *equity analyses *describing the distributional impact of interventions [9-12]. These studies aim to analyze to the extent interventions reach and benefit disadvantages groups, such as the poor or certain ethnicities, or otherwise vulnerable populations.

## The need for multi-criteria decision analysis

However, the above approaches offer limited guidance to policy makers in their choice of interventions, for a number of reasons. Firstly, they were developed in isolation from each other, and concentrate on single criteria for priority setting – be it effectiveness, cost-effectiveness, burden of disease, or equity analysis, and do not advice on how to integrate or judge the relative importance of each criterion. In reality, policy makers need to make choices on interventions taking those criteria into account simultaneously. Moreover, criteria can easily conflict. For example, interventions targeting marginalized populations in remote areas of a country are likely to be more costly and therefore less cost-effective than those covering only people in urban areas [36]. Also, not all criteria are equally important: depending on the pro-poor stance of a country, policy makers may value interventions that target the poor more highly than those that stimulate economic growth.

Secondly, these approaches do not cover all criteria that are relevant to policy makers. For example, they are not able to capture preferences of society regarding 'the rule of rescue' in acute cure or regarding interventions related to irresponsible behavior of patients. A further complicating factor is that prioritisation decisions typically draw upon multidisciplinary knowledge bases, incorporating clinical medicine, public health, social sciences and ethics, and policy makers lack expertise to adequately interpret on all these aspects.

As a result, policy makers may not be able to utilize all available and necessary information in choosing between different interventions, and priority setting is ad-hoc (Figure 1). This stresses the need for the scientific development of MCDA to support priority setting, which has recently indeed been identified as one of the most important issues in health system research [5]. Baltussen and others have argued that MCDA should allow a trade-off between various criteria, and should establish the relative importance of criteria in a way that allows a rank ordering of a comprehensive set of interventions [4,37] (Figure 1). The underlying idea is that policy makers fund interventions according to this rank ordering until their budget is exhausted.

## Methods of multi-criteria decision analysis

In stark contrast with the near-absence of applications of MCDA to allocation decisions in health care is the widespread acceptance and routine use of MCDA in other disciplines, e.g. to structure remedial decisions at contaminated sites in environmental sciences [38]. MCDA has also been applied in agricultural [39], energy [40], and marketing [41] sciences. In those disciplines, MCDA has evolved as a response to the observed inability of people to effectively analyze multiple streams of dissimilar information. The analysis establishes preferences between options by reference to an explicit set of objectives that the decision making body has identified, and for which it has established measurable criteria to assess the extent to which the objectives have been achieved [42]. MCDA offers a number of ways of aggregating the data on individual criteria to provide indicators of the overall performance of options.

This section outlines the main principles of MCDA, heavily drawing on standard works in those disciplines [42-45]. Wherever we use to term 'option' in this paper, this refers to 'intervention' in the context of priority setting in health, and the terms are used interchangeably. It first presents the performance matrix, which is a standard feature of every multi-criteria analysis. Next, it explains how the basic information in the performance matrix can be processed – either qualitatively or quantitatively.

### The performance matrix

In a performance matrix, each row describes an option and each column describes the performance of the options against each criterion. The criteria are the measures of performance by which the options will be judged, and must be carefully selected, to assure completeness, feasibility, and mutual independence, and avoid redundancy and an excessive number of criteria. The individual performance assessments are often qualitative descriptions, or natural units, or sometimes a (crude) numerical scale [42]. Table 1 shows a simplified example, on the basis of the performance of a number of different interventions in regard to a set of criteria thought to be relevant in policy making. These criteria are cost-effectiveness, severity of disease, whether a disease is more among the poor, and age. As can be seen, some of these criteria are measured on a binary scale (a tick indicates a disease is more prevalent among the poor than among the rich), nominal scale (age), ordinal scales (severity of disease), or ratio scale (cost-effectiveness).

**Table 1:** Performance matrix

Options	Cost-effectiveness	Severity of disease	Disease of the poor	Age
Antiretroviral treatment in HIV/AIDS	US$200 per DALY	●●●●	√	15 years and older
Treatment of childhood pneumonia	US$20 per DALY	●●●●	√	0–14 years
Inpatient care for acute schizophrenia	US$2000 per DALY	●●		15 years and older
Plastering for simple fractures	US$50 per DALY	●		all

A tick indicates the presence of a feature. Severity of disease is shown of a four-star scale, with more stars indicating a more severe disease.

### Qualitative analysis of the performance matrix

The performance matrix may be the final product of the analysis, allowing the decision maker to qualitatively rank the options. Such intuitive processing of the data can be quick and effective, but it may also lead to the use of unjustified assumptions, causing incorrect ranking of options [42]. The decision maker can come to a few types of comparisons.

#### Dominance

Direct inspection of the performance matrix can show if any of the options are dominated by others. Dominance occurs when one option performs at least as well as another on all criteria and strictly better than the other on at least one criterion. In practice, dominance is likely to be rare, and the extent to which it can help to discriminate between many options and to support real decisions is correspondingly limited.

#### Subjective interpretation

Decision makers may also use the performance matrix to add recorded performance levels across the rows (options) to make some holistic judgment between options about which ones are better. However, this implies that all criteria contribute with equal importance to options' overall performance, when this has not been established. More generally, a subjective interpretation of the matrix is prone to many well-documented distortions of human judgments [6,7]. In marketing, this method is also called the 'pros and cons' or 'balance sheet' analysis, and is used by salespeople to gain commitment from a buyer by asking to think of the pros and cons of various alternatives [41].

### Quantitative analysis of the performance matrix

In analytically more sophisticated MCDA techniques the information in the basic matrix is usually converted into consistent numerical values. The key idea is to construct scales representing preferences for the consequences, to weight the scales for their relative importance, and then to calculate weighted averages across the preference scales [42].

First, the expected consequences of each option are assigned a numerical score reflecting the strength of preference scale for each option for each criterion. More preferred options score higher on the scale, and less preferred options score lower. The scoring can be based on a value function, which translates a measure of achievement on the criterion in to a value score on the scale. Alternatively, when a commonly agreed scale of measurement does not exist, direct rating can be used and is based on the judgment of an expert simply to associate a number on that scale with the value of each option on that criterion. Or, scores can be obtained by eliciting from the decision maker a series of verbal pair wise assessments expressing a judgment of the performance of each option relative to each of the others (e.g. the Analytical Hierarchy Process does this (see below)). The scores are presented in Table 2 in normal figure.

**Table 2:** Scoring the options.

Options	Cost-effectiveness	Severity of disease	Disease of the poor	Age	**Total**
Antiretroviral treatment in HIV/AIDS	50	100	100	0	**70**
Treatment of childhood pneumonia	100	100	100	100	**100**
Inpatient care for acute schizophrenia	0	50	0	0	**5**
Plastering for simple fractures	100	25	0	50	**48**

**Weights**	**40**	**10**	**40**	**10**	

Preference scores for 'cost-effectiveness' are obviously inverse to its values, and are based on three categories: it scores 0 if the cost-effectiveness is higher than US$300 per DALY, 50 if between US$100 and US$300, and 100 if below US$100 per DALY. For 'disease of the poor', if the feature is present, it scores 100, otherwise 0. Preference scores for 'severity of disease' are scaled between 0 and 100 in proportion to their stars. Assuming decision makers have a preference to treat young people over old, '0–14 years' receives a score of 100, '15 years and older' a score of 0, and 'all ages' a score of 50. Preference scores are presented here for illustrative purposes only, and are arbitrary.

Second, numerical weights are assigned to define, for each criterion, the relative valuations of a shift between the top and bottom of the chosen scale. Weights can be obtained by comparing weights of criterions to the most important criterion, e.g. on the basis of group discussions. In a next step, those weights are calculated to sum up to 100 in total. In the example in Table 2, weights are presented in bold figure: 'cost-effectiveness' and 'disease of the poor' are both assigned a value of 40, and the other criteria a value of 10.

Mathematical routines then combine these two components to give an overall assessment of each option being appraised. At this stage, it is important to determine whether trade-offs between different criteria are acceptable, so that good performance on one criterion can in principle compensate for weaker performance on another. Most public decisions admit such trade-offs, but there may be some circumstances, perhaps where ethical issues are central, where trade-offs of this type are not acceptable. If it is not acceptable to consider trade-offs between criteria, then there are a limited number of non-compensatory MCA techniques available [42]. Where compensation is acceptable, and low scores on one criterion may be compensated by high scores on another, compensatory MCA techniques are used that involve aggregation of each option's performance across all the criteria to form an overall assessment of each option, on the basis of which the set of options can be compared. These techniques are usually based on multi-attribute utility theory [46]. The principal difference between the main families of MCA methods is the way in which this aggregation is done.

#### The simple linear additive evaluation model

If it can either be proved, or reasonably assumed, that the criteria are preferentially independent of each other, then the simple linear additive evaluation model is applicable. The linear model shows how an option's values on the many criteria can be combined into one overall value. This is done through multiplication of the value score on each criterion by the weight of that criterion, and then adding all those weighted scores together. For example, in Table 2, antiretroviral treatment in HIV/AIDS scores 50 on the criterion 'cost-effectiveness', and the weight of that criterion is 40/100: the weighted score is then 50 * 40/100 = 20. In a similar way, the weighted scores on 'severity of disease', 'disease of the poor', and 'age' are respectively 10, 40, and 0. The weighted scores sum up to 70, which is shown in the final column. Treatment of childhood pneumonia has a total score of 100, and is therefore the preferred option, followed by antiretroviral treatment in HIV/AIDS, plastering for simple fractures (48), and inpatient care for acute schizophrenia (5).

#### The analytical hierarchy process

The analytic hierarchy process also develops a linear additive model, but, in its standard format, uses procedures for deriving the weights and the scores achieved by alternatives, which are based, respectively, on pair wise comparisons between criteria and between options. Thus, for example, in assessing weights, the decision maker is asked a series of questions, each of which asks how important one particular criterion is relative to another for the decision being addressed.

#### Outranking methods

A rather different approach depends upon the concept of outranking, and seeks to eliminate alternatives that are, in a particular sense, 'dominated'. However, unlike the straightforward dominance idea outlined above, 'outranked dominance' gives more influence to some criteria than others. One option is said to outrank another if it outperforms the other on enough criteria of sufficient importance (as reflected by the sum of the criteria weights) and is not outperformed by the other option in the sense of recording a significantly inferior performance on any one criterion. The outranking concept indirectly captures some of the political realities of decision-making, by downgrading options that perform badly on any one criterion (which might in turn activate strong lobbying from concerned parties and difficulty in implementing the option in question). In the example, in Table 1, all interventions are outranked by 'treatment of child pneumonia', and this illustrates its low discriminative power and hence its limited potential for priority setting, especially in the context of many criteria and many interventions.

## Applications to health care

To date, MCDA knows very few applications to guide resource allocation decisions in health care, in either western or developing countries. These applications have used MCDA to different extents: to only illustrate its principles, to identify the criteria for priority setting, to identify and weigh the criteria for priority setting, or more comprehensive approaches that result in a rank ordering of interventions.

James et al. [47] illustrated the *principles of MCDA *by demonstrating the potential impact of alternative weights for equity and efficiency criteria on the ranking of a number of hypothetical interventions.

The *criteria *for priority setting were identified by two merely qualitative studies in Uganda [4,48], including medical (e.g. effectiveness, cost-effectiveness, quality of evidence, severity of disease) and non-medical criteria (e.g. age, gender, and area of residence). Yet, they did not establish the weights of these criteria in a way that allows a rank ordering of interventions. Recently, a number of tools have been developed that take into account various criteria, but these do not explicitly attach weights to these criteria. Tugwell et al. [49] have proposed the 'equity effectiveness loop' to highlight equity issues inherent in assessing health needs, effectiveness and cost-effectiveness of interventions. The 'marginal budgeting for bottlenecks' tool aims to bridge between costing, cost-effectiveness and burden of disease analysis [50]. 'District health accounts' is a tool designed to help districts analyze their budgets and expenditures so that budgets can be set against priorities as defined by the prevailing burden of disease, and as such integrates budgeting, costing and burden of disease analysis [51]. In the Netherlands, Dunning identified a number of criteria for public reimbursement of health care. However, some of its criteria – especially medical need – were not well defined, and its application therefore suboptimal [52].

Further studies have quantified the *scores and weights of criteria*, but these are typically limited to two criteria only: e.g. on cost-effectiveness and equity [53], or on age and severity of illness [54,55].

Recently, two comprehensive MCDA approaches have been developed. Wilson et al. [56] developed a prioritization framework in an English Primary Care Trust. Through group discussion with policy makers, a number of *criteria *were identified (such as effectiveness, quality of life, access/equity, need, and prevention) and were *weighed *into four broad 'levels of importance'. Next, the groups *scored *four hypothetical interventions on those criteria on a scale from 0–10. A simple linear additive evaluation model was used to calculate overall scores, and interventions were rank ordered according to their 'cost-value' ratio (estimated by dividing the costs of an interventions by the overall score). The authors consider the framework as a promising tool for prioritizing interventions in the Primary Care Trust.

**Figure 2:**
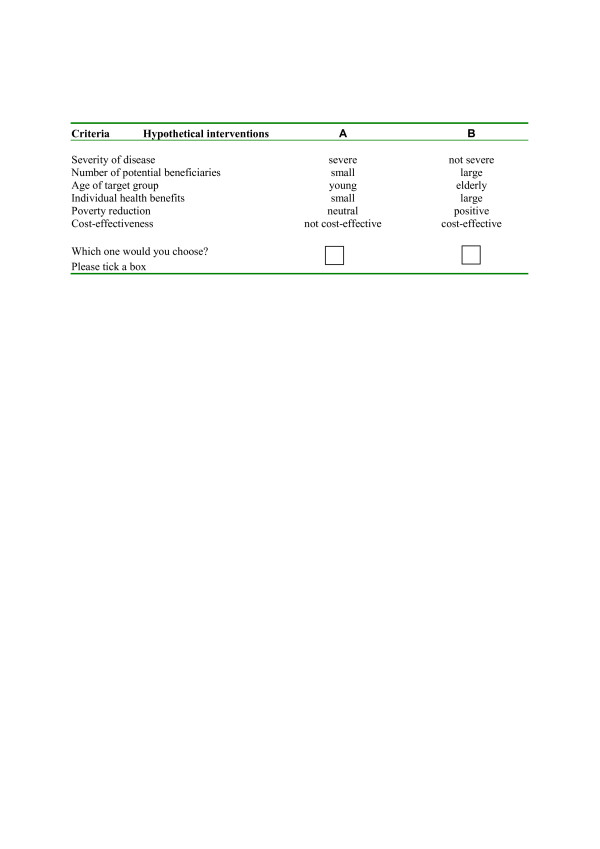
Example of a question in a discrete choice experiment.

Baltussen et al. carried out explorative research to prioritize health interventions in Ghana and Nepal using discrete choice experiments [37,57]. In Ghana, *criteria *were identified through a series of group discussions with policy makers, and included 'cost-effectiveness', 'poverty reduction', 'age', 'severity of illness', 'budget impact' and 'burden of disease'. *Intervention scores *on those criteria were based on poverty profiles, burden of disease and cost-effectiveness analysis as presented in the World Health Report 2002 [58], and were expressed on a binary scale with arbitrary cut-off values. The *relative weights *of the various criteria were estimated through the use of discrete choice experiments (DCE) [59], with a large number of policy makers. In the DCE, respondents choose their preferred option from sets of hypothetical interventions, each consisting of a bundle of criteria that described the intervention in question, with each criterion varying over a range of scores (Figure 2). The criteria were constant in each scenario, but the scores that described each criterion varied across interventions. Analysis of the options chosen by respondents in each set revealed the extent to which each criterion was important. The work in Ghana showed that policy makers give high value to interventions that are cost-effective (score of 1.42), reduce poverty (1.25), target the young (0.84), or target severe diseases (0.38). Using a simple linear additive evaluation model, total scores were calculated for a set of interventions, and rank ordered accordingly: high priority interventions in Ghana were prevention of mother to child transmission in HIV/AIDS control, and treatment of pneumonia and diarrhea in childhood. Lower priority interventions were certain interventions to control blood pressure, tobacco and alcohol abuse. Full details are reported elsewhere [37].

## Conclusion

This paper has shown the basic principles of MCDA, and the need for its application in health. Whereas decisions in health care are often characterized by informal judgment unsupported by analysis, MCDA may be an important tool towards a more rational priority setting process.

This paper has introduced various approaches to MCDA, and these are mainly characterized by how the performance matrix is interpreted. Some approaches seem more useful to prioritise health interventions than others. First, the priority setting process involves many criteria and many interventions, and since intuitive processing of this complex data can lead to unjustified conclusions, quantitative rather than qualitative analyses seem apt. Second, compensatory rather than non-compensatory techniques seem apt as public decisions typically allow trade-offs between criteria (perhaps except in situations where ethical issues are central). Third, because of the need to rank order a large number of interventions rather than to identify a single (or small number of) dominant interventions, the linear additive model seems more suitable than the outranking method. As noted above, first experiments with the linear additive model have been carried out in Ghana and Nepal [37,55], and encouraging results indicate the potential of the approach to inform policy makers on actual priority setting of interventions.

This paper has illustrated the use of MCDA with some simplified examples. In a practical application, interventions may be need evaluated at different geographic coverage levels, to inform decisions on the choice between scaling up existing interventions, or implementing new interventions. WHO-CHOICE does evaluate interventions at coverage levels of 50%, 80%, and 95% for this purpose [60,61]. In addition, interventions may need to be evaluated not only in isolation, but also in combination, since interactions may exist between interventions in either costs and/or effects. For this reason, WHO-CHOICE does evaluate interventions in isolation and in combination [62].

The priority setting process should be strongly embedded in the organizational context, probably with a central role for an advisory panel [63]. An advisory panel comprises key stakeholders such as health personnel, policy makers, finance and information staff, and community representatives. The panel has an important role in the definition of the relevant criteria and their relative importance for priority setting, and making recommendations for reallocating resources on the basis of MCDA results. In the latter, the advisory panel may diverge from MCDA results because of e.g. pragmatic considerations. In other words, while MCDA suggests a rank ordering of interventions, this not necessarily means that interventions should be funded accordingly till the budget is exhausted. This is based on the notion that MCDA should not be seen as a formulaic or technocratic approach to priority setting, but rather as an aid to policy making.

MCDA will contribute to the fairness of the priority setting process. According to Daniels and Sabin's ethical framework of accountability for reasonableness, priority setting is said to be fair if the priority setting process, decisions and rationales are accessible and relevant; and an appeals and enforcement mechanism are established [64]. MCDA contributes to the first two conditions because of its systematic and transparent nature.

We call for a shift away from present tools for priority setting – that tend to focus on single criteria for priority setting – towards transparent and systematic approaches that take into account all relevant criteria simultaneously. Although very little work has been done so far on comprehensive MCDA approaches, a number of tools that aim to bridge the different analytical approaches are being developed. It is time to assess the current state of the art of the methods, and to stimulate the development of a new generation of more evidence-based priority setting tools.
